# Relationship between Optical Coherence Tomography and Electrophysiology of the Visual Pathway in Non-Optic Neuritis Eyes of Multiple Sclerosis Patients

**DOI:** 10.1371/journal.pone.0102546

**Published:** 2014-08-28

**Authors:** Prema Sriram, Chenyu Wang, Con Yiannikas, Raymond Garrick, Michael Barnett, John Parratt, Stuart L. Graham, Hemamalini Arvind, Alexander Klistorner

**Affiliations:** 1 Australian School of Advanced Medicine, Macquarie University, Sydney, Australia; 2 Brain and Mind Research Institute, University of Sydney, Sydney, Australia; 3 Concord Hospital, Sydney, Australia; 4 St Vincent’s Hospital, Sydney, Australia; 5 Royal North Shore Hospital, Sydney, Australia; 6 Save Sight Institute, Department of Ophthalmology, University of Sydney, Sydney, Australia; Institute Biomedical Research August Pi Sunyer (IDIBAPS) - Hospital Clinic of Barcelona, Spain

## Abstract

**Purpose:**

Loss of retinal ganglion cells in in non-optic neuritis eyes of Multiple Sclerosis patients (MS-NON) has recently been demonstrated. However, the pathological basis of this loss at present is not clear. Therefore, the aim of the current study was to investigate associations of clinical (high and low contrast visual acuity) and electrophysiological (electroretinogram and multifocal Visual Evoked Potentials) measures of the visual pathway with neuronal and axonal loss of RGC in order to better understand the nature of this loss.

**Methods:**

Sixty-two patients with relapsing remitting multiple sclerosis with no previous history of optic neuritis in at least one eye were enrolled. All patients underwent a detailed ophthalmological examination in addition to low contrast visual acuity, Optical Coherence Tomography, full field electroretinogram (ERG) and multifocal visual evoked potentials (mfVEP).

**Results:**

There was significant reduction of ganglion cell layer thickness, and total and temporal retinal nerve fibre layer (RNFL) thickness (p<0.0001, 0.002 and 0.0002 respectively). Multifocal VEP also demonstrated significant amplitude reduction and latency delay (p<0.0001 for both). Ganglion cell layer thickness, total and temporal RNFL thickness inversely correlated with mfVEP latency (r = −0.48, p<0.0001 respectively; r = −0.53, p<0.0001 and r = −0.59, p<0.0001 respectively). Ganglion cell layer thickness, total and temporal RNFL thickness also inversely correlated with the photopic b-wave latency (r = −0.35, p = 0.01; r = −0.33, p = 0.025; r = −0.36, p = 0.008 respectively). Multivariate linear regression model demonstrated that while both factors were significantly associated with RGC axonal and neuronal loss, the estimated predictive power of the posterior visual pathway damage was considerably larger compare to retinal dysfunction.

**Conclusion:**

The results of our study demonstrated significant association of RGC axonal and neuronal loss in NON-eyes of MS patients with both retinal dysfunction and post-chiasmal damage of the visual pathway.

## Introduction

Susceptibility of the visual system to damage in multiple sclerosis (MS) is well documented. Apart from acute inflammation of the optic nerve, which is often the first manifestation of the disease, all other elements of the visual pathway from outer-retina to visual cortex are frequently involved.

Hierarchical organization of the visual system coupled with recent technological advances makes visual pathway an ideal model to study mechanisms of MS. Retinal Ganglion Cells (RGC) are of particular interest since their unique position and accessibility to direct *in vivo* measurement by high resolution spectral domain OCT allows study of MS-related neurodegeneration including the possible effect of pathological changes in neighboring cellular elements, which are yet to be characterised.

It is well recognised that axonal transection during acute inflammation of the optic nerve (optic neuritis) is a major cause of RGC axonal and neuronal loss in MS. Correlation of RNFL thickness with stage of MS, brain atrophy, degree of disability and disease duration found in a number of cross-sectional studies incited considerable interest in using assessment of the anterior visual pathway as a structural marker of CNS neurodegeneration in MS [1]–[8] and was even suggested as a possible outcome for future neuroprotection trials. [4], [9], [10] Recently, however, loss of RGC has also been demonstrated in non-optic neuritis (NON) eyes. A meta-analyses published by Petzold et al [11] showed significant thinning of RGC axons (so called retinal nerve fiber layer-RNFL) in MS-NON eyes. However, the pathological basis of this loss at present is not clear.

In the current study we performed functional assessment of the visual pathway in NON-eyes of MS patients using clinical (high and low contrast visual acuity) and electrophysiological (electroretinogram and multifocal Visual Evoked Potentials) measures and its relationship with RGC. We hypothesized that studying potential associations of functional measures with neuronal and axonal loss of RGC may advance our understanding of the nature of this loss.

## Methods

Sixty-two patients with relapsing remitting multiple sclerosis with no previous history of optic neuritis in at least one eye were enrolled. Patients with any other systemic or ocular disease that could confound results, such as diabetes, retinal lesions or glaucoma, were excluded.

Latency of the mfVEP demonstrated significant inverse correlation with GCL thickness, global and temporal RNFL thickness (r = −0.48, p<0.0001; r = −0.53, p<0.0001 and r = −0.59, p<0.0001 respectively) (Fig. 3a–c).

### Ethics statement

The Institutional Review Board of University of Sydney and Macquarie University approved the study. Procedures followed the tenets of the Declaration of Helsinki and written informed consent was obtained from all participants.

### Clinical assessments

Best-corrected visual acuity (VA) was measured using Sloan high contrast (100%) and low contrast letter acuity charts (LCVA) (2.5% and 1.25%) at 4 m. Snellen VA equivalents (documented in LogMAR notation) were determined from 100% contrast charts. For LCVA, the numbers of letters correctly identified (maximum 60/chart) were recorded for each eye. A detailed ophthalmological examination was also performed.

In addition, amplitude of the mfVEP significantly correlated with tRNFL and RGC layer thickness (r = 0.44, p = 0.002 and r = 0.32, p = 0.026) and displayed tendency for association with total RNFL (r = 0.26, p = 0.057) (Fig. 4a–c).

GCL thickness, global and temporal RNFL thickness also inversely correlated with the photopic b-wave latency (r = −0.35, p = 0.01; r = −0.33, p = 0.025; r = −0.36, p = 0.008 respectively) (Fig. 5a–c). No correlation with other ERG parameters was noted.

### mfVEP recording and analysis

Multifocal VEP testing was performed using the Accumap (ObjectiVision Pty. Ltd., Sydney, Australia) employing standard stimulus conditions that entailed recordings from 58 segments of the visual field. Monocular recordings were completed for 10 to 12 runs until a sufficient signal to noise ratio was reached. Four gold cup electrodes were placed around the inion and used for bipolar recording from four channels: superior and inferior; left and right, and obliquely between horizontal and inferior electrodes. Data were analysed using Opera V1.3 software. For amplitude analysis the largest peak-trough amplitude within the interval of 70–200 ms was determined. The second peak of the wave of maximum amplitude for each segment in the visual field was used for latency analysis. Averaged (across entire stimulated field) amplitude and latency were used for analysis. mfVEP measurements were compared to values of 25 age and gender matched controls.

### Full-field ERG

Full-field ERG was performed according to the ISCEV standard [12] using ESPION system (Diagnosys LLC, Lowell, MA, USA). Amplitude and latency of dark-adapted rod response, dark-adapted mixed rod/cone response at 2 levels of flash intensity and light adapted cone response were analysed.

### Optical coherence tomography (OCT)

Optical Coherence Tomography was performed on a Spectralis scanner (Heidelberg Engineering). Global RNFL (gRNFL) thickness and temporal quadrant RNFL (tRNFL) thickness were assessed using the RNFL protocol. In addition, a radial protocol using a star-like pattern of line scans centered on the macula with resolution of 1536 pixels was used for measurement of thickness of retinal layers. Analysis was performed on vertical scan only. One hundred scans were averaged for each line scan. Thirty degrees of visual angle (15 degrees of eccentricity) were scanned, but only the central 14 degrees (7 degrees of eccentricity) were used for analysis, since the definition of layers becomes much less distinct beyond that. Retinal layers were segmented automatically using a custom designed algorithm, which applied vessel detection and removal, multiple size median filtering, and Canny edge detection to identify borders of retinal layers [13].

**Figure 1:**
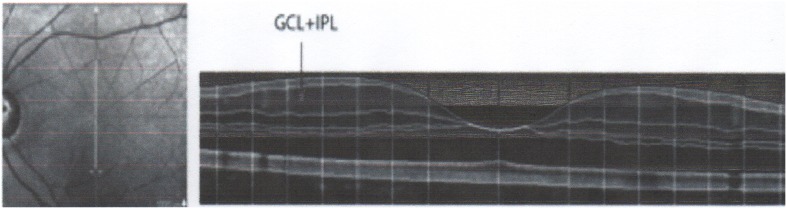
OCT scanning pattern (left) and segmentation of retinal layers (right).

The Ganglion Cell Layer and Inner Plexiform layer were combined together (for brevity this layer will be called GCL) (see example in Fig. 1). The thickness of this layer was measured at seven points for each hemifield, which were equally distributed between 1.75 and 7 degrees of eccentricity. OCT measurements were compared to values of 50 age and gender matched controls.

### Statistical Analyses

Statistical analyses were performed using IBM SPSS 20. Pearson correlation coefficient was used for bivariate correlation, while Student’s t-test was used to compare means. Significance was determined at 0.05 level.

For the multivariate linear regression model, a backward elimination variable selection procedure in which all variables are entered into the equation and then sequentially removed based on removal criteria, was employed. Probability of F = 0.05 was used as entry criteria, while F = 0.1 was used as removal criteria.

All procedures followed the tenets of the Declaration of Helsinki and written informed consent was obtained from all participants.

## Results

In total 62 RRMS patients were recruited. Four patients had high myopia and had to be excluded from analysis. One patient had an extremely large optic disc and hence his temporal RNFL was not included in analysis. Therefore, data from 58 patients (39.9±11.3 years, 18 Males/40 Females) were analyzed. Average time from diagnosis of MS was 4.7±2.9 years (1–14 years). Twenty-five patients had a previous history of optic neuritis in one eye only. Thirty-three patients did not have a history of optic neuritis in either eye. One eye of these patients was randomly selected and analysed together with the fellow eyes of optic neuritis patients.

There was significant reduction of GCL thickness as well as total and temporal RNFL in the NON-eyes of MS patients (Table 1). All three measures correlated between each other (p<0.001 for all pairs). In relation to normal controls tRNFL demonstrated by far the largest thinning as compared to gRNFL and RGC thickness (10%, 5.9% and 6.3% for tRNFL, gRNFL and GCL respectively).

**Table 1:** Comparison of functional and structural measurements in controls and MS-NON eyes.

	Control (n = 25)	MS-NON eyes (n = 58)	MS-NON eyes (n = 58)
	Mean±SD	Mean±SD	p value*
Global RNFL (µ)	99.2±7.5	93.6±9.9	0.002
Temp RNFL (µ)	70.8±7.8	64.2±9.3	0.0002
GCL (µ)	86.5±5.5	81.4±7.1	<0.0001
mfVEP amplitude (µV)	238.1±36.1	151.6±42.9	<0.0001
mfVEP latency (µV)	149.3±5.1	161.5±9.2	<0.0001
Dim white b-wave amplitude (µV)	354.4±134.8	353.7±90.7	0.92
Dim white b-wave latency (ms)	97.7±8.9	97.3±7.9	0.89
Dark max 3 a-wave amplitude (µV)	−268.3±48.4	−287.0±58.8	0.11
Dark max 3 a-wave latency (ms)	16.6±1.4	16.8±0.6	0.37
Dark max 3 b-wave amplitude (µV)	490.9±103.8	524.5±104.6	0.10
Dark max 3 b-wave latency (ms)	53.1±3.6	53.9±4.0	0.26
Dark max 12 a-wave amplitude (µV)	−325.5±52.4	−342.5±64.6	0.20
Dark max 12 a-wave latency (ms)	13.5±1.1	13.7±0.9	0.43
Dark max 12 b-wave amplitude (µV)	511.9±109.2	537.0±111.1	0.27
Dark max 12 b-wave latency (ms)	52.9±1.9	53.4±1.5	0.17
Photopic a-wave amplitude (µV)	−41.4±18.1	−46.3±10.9	0.15
Photopic a-wave latency (ms)	15.1±0.8	15.4±0.6	0.06
Photopic b-wave amplitude (µV)	174.7±35.5	185.3±41.4	0.21
Photopic b-wave latency (ms)	29.8±0.8	30.4±0.8	0.004

*p value calculated using student t-test.

**Figure 2:**
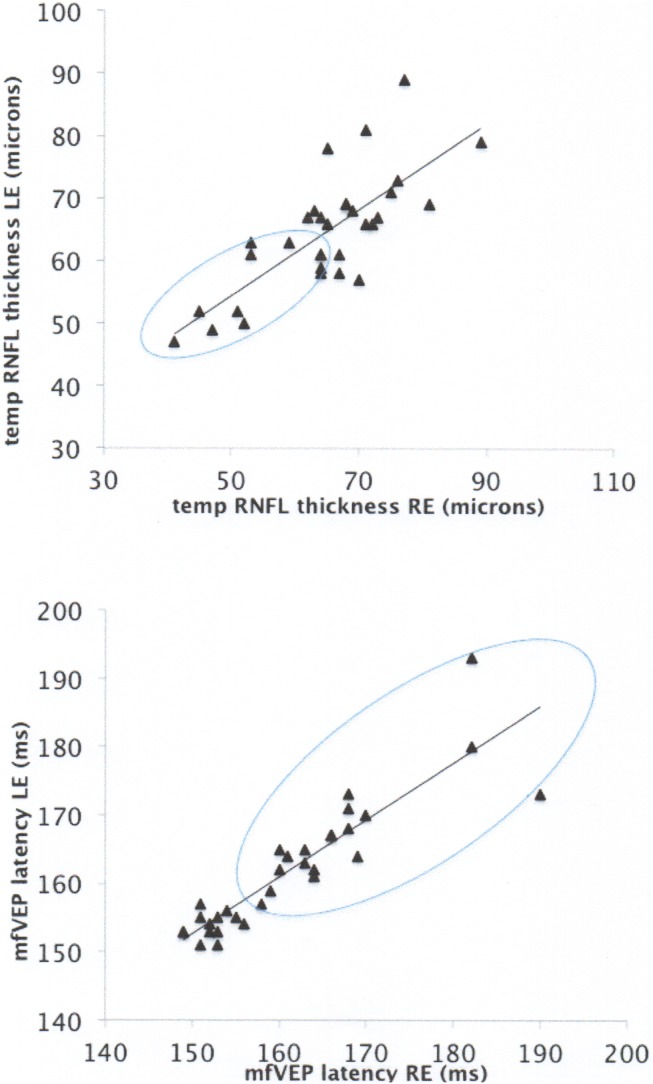
Figure 2a: Correlation of temporal RNFL thickness between the right and left eyes in MS patients without ON in either eye. Figure 2b: Correlation of mfVEP latency between the right and left eyes in MS patients without ON in either eye.

In patients without history of ON in either eye RNFL and RGC thinning, where present, tended to be binocular (see example of tRNFL in Fig. 2a, oval includes points below 5^th^ percentile (1.96 SD) of tRNFL thickness in normal controls). To quantify binocular nature of RGC axonal and neuronal loss we performed correlation between study eye and non-study eye for tRNFL, gRNFL and RGC thickness. To avoid effect of individual inter-eye correlation only eyes with significantly thinner retinal layers (below 5^th^ percentile of thickness value of the normal controls) were included. Correlation was highly significant (p<0.001 for all).

**Figure 3:**
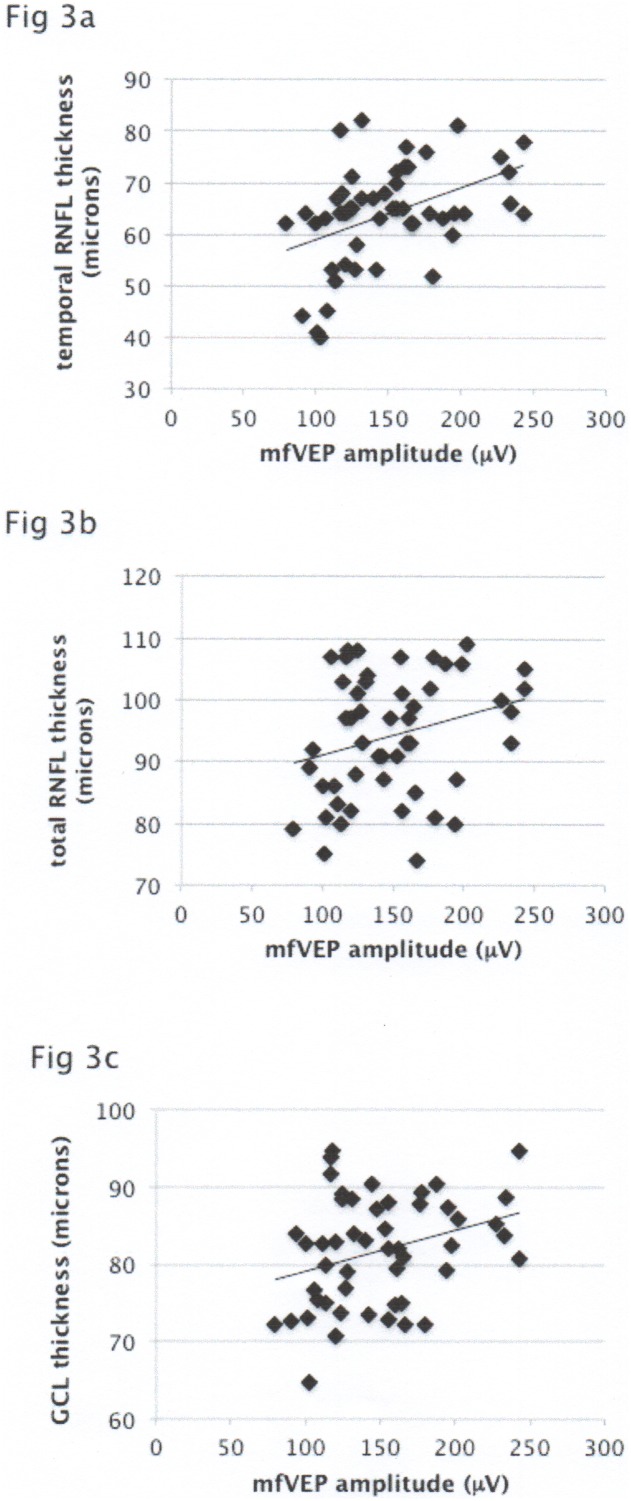
Figure 3a: Correlation of mfVEP latency with temporal RNFL thickness. Figure 3b: Correlation of mfVEP latency with total RNFL thickness. Figure 3c: Correlation of mfVEP latency with GCL thickness.

**Figure 4:**
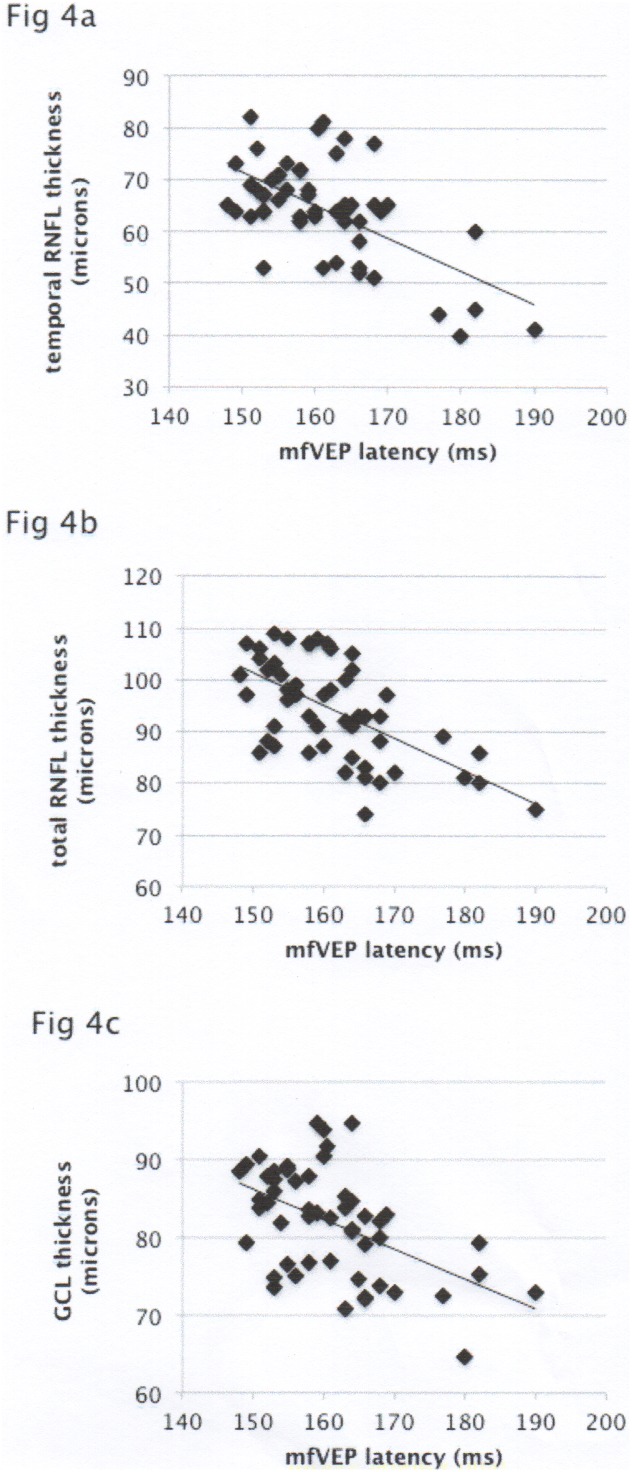
Figure 4a: Correlation of mfVEP amplitude with temporal RNFL thickness. Figure 4b: Correlation of mfVEP amplitude with total RNFL thickness. Figure 4c: Correlation of mfVEP amplitude with GCL thickness.

**Figure 5:**
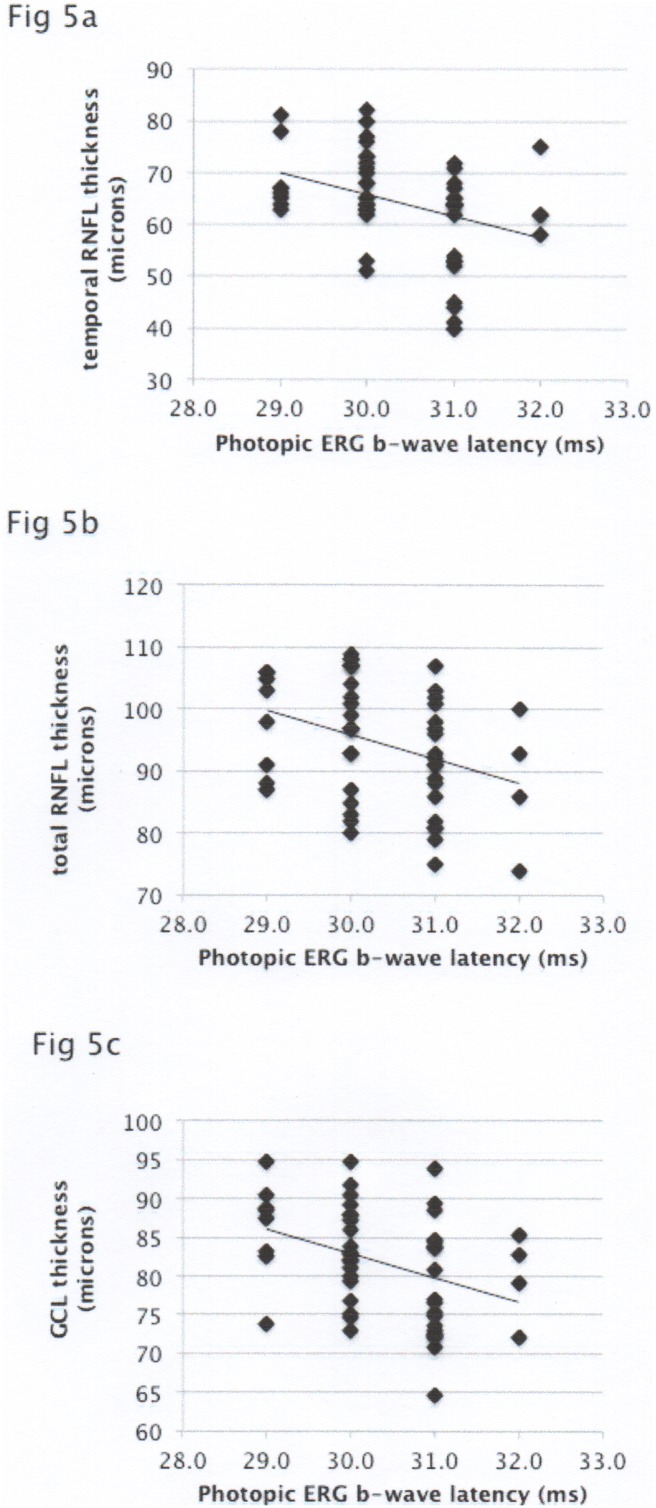
Figure 5a: Correlation of photopic ERG b-wave latency with temporal RNFL thickness. Figure 5b: Correlation of photopic ERG b-wave latency with total RNFL thickness. Figure 5c: Correlation of photopic ERG b-wave latency with GCL thickness.

Multifocal VEP demonstrated significant amplitude reduction and latency delay in the MS-NON eyes as compared to normal controls (p<0.0001 for both) (Table 1).

In patients without ON in either eye the mfVEP latency delay, where present, also displayed tendency for being binocular (Fig. 2b, oval indicates points above 5% (1.96 SD) of mfVEP latency in normal controls).

The b-wave latency of the photopic ERG was significantly delayed in the MS-NON eyes in comparison to the normal controls (p = 0.03). No other ERG parameters were affected (Table 1).

LCVA (both 2.5% and 1.25% contrast) significantly correlated with global and temporal RNFL thickness as well as mfVEP amplitude and inversely correlated with mfVEP latency (Table 2). No correlation between high contrast visual acuity and any of the measures was found.

**Table 2:** Correlation with LCVA.

	LCVA 2.5% contrast	LCVA 2.5% contrast	LCVA 1.25% contrast	LCVA 1.25% contrast
	Correlation (r)	p value	Correlation (r)	p value
Global RNFL	0.44	0.003	0.4	0.008
Temporal RNFL	0.39	0.01	0.32	0.03
mfVEP amplitude	0.47	0.002	0.36	0.02
mfVEP latency	−0.39	0.01	−0.41	0.007

There were no correlations between ERG and mfVEP parameters.

### Multivariate linear regression model

Since RNFL and RGC layer thinning correlated with both photopic ERG latency and mfVEP latency we used multivariate linear regression model to assess combined predictive power and relative strength of those association. GCL thickness, gRNFL and tRNFL were used as dependent variables in individual models. The latency of mfVEP, the photopic b-wave latency of ERG, age, gender and disease duration were entered into each model.

Individual models explained 47% of tRNFL thickness variability, 36% of gRNFL thickness variability and 30% of GCL thickness variability. Multifocal VEP latency was significant in all models, while the photopic b-wave latency contributed significantly to tRNFL and GCL models. No other variables were retained by any of the models. According to Standardized Beta coefficient, the mfVEP latency was a much stronger predictor of RNFL and RGC layer thinning compared to the photopic ERG b-wave latency (Table 3).

**Table 3:** Linear Regression Model.

	GCL	GCL	Global RNFL	Global RNFL	Temporal RNFL	Temporal RNFL
Variables	Stand Beta	Sig	Stand Beta	Sig	Stand Beta	Sig
mfVEP latency	−0.46	<0.001	−0.61	<0.001	−0.56	<0.001
Photopic b-wave ERG latency	−0.25	0.04			−0.24	0.03

## Discussion

In the current study we assessed functional measures of the visual pathway in NON-eyes of MS patients and its association with axonal and neuronal loss of RGC.

Our result confirms previous reports of RNFL and RGC layer thinning in NON-eyes of MS patients as compared to normal controls. While this reduction was significant, it was considerably less then the loss typically reported in ON eyes. Association of thinner RNFL and RGC thickness with worse LCVA score demonstrated potential functional significance of this loss. It has been shown that conventional measures of the visual function suffer only after RNFL loss reaches a certain threshold (<70 µ). [14] Our study, however, shows that even minor losses of RGC and their axons may result in measurable deterioration of vision, provided sensitive means for the assessment of the visual function, such as LCLA, are used [15].

Correlation of RNFL and RGC thickness with amplitude of the mfVEP, which is regarded as an objective marker of the visual function, also supports functional significance of RGC neurodegeneration in NON-eyes.

RNFL thinning in NON eyes has previously been attributed to several factors, such as sub-clinical inflammation of the optic nerve, primary retinal degeneration or trans-synaptic transmission of the damage from the posterior visual pathway. [2], [4], [16], [17] Therefore, it is of interest that in the patients without history of ON in either eye, reduction of both RNFL and RGC thickness displayed binocular nature. Due to the fact that ON fibers are partially crossing at the chiasm, this may suggest retro-chiasmal origin of the loss.

On a potential abnormality of the retro-chiasmal pathway in our cohort also pointed out binocular nature of mfVEP delay. While VEP latency delay in NON-eyes of MS patients has been reported in numerous studies, binocular character of this delay has only recently been noticed. [17] Correlation performed between eyes with delayed latency only, performed in the current study, confirmed binocular character of the delay even when inter-subject variability was minimized.

VEP delay not only showed similar pattern of binocular abnormality in patients without episode of ON in either eye, but more importantly, it demonstrated significant correlation with RNFL and RGC layer thinning in entire study cohort.

It is believed that VEP is generated at the level of striate cortex by the combined activity of post-synaptic potentials. [18], [19] Therefore, VEP latency may be affected by demyelinating process along the retro-chiasmal part of the visual pathway, namely optic tract (OT) and optic radiation (OR). Since the OT represents continuation of the RGC axons after chiasmal crossing, the lesions of the OT can cause RNFL and RGC layer thinning via the mechanism of retrograde degeneration. While there are reported cases of optic tract lesions in MS (which typically presented with homonymous visual field defect), those lesions are rare. [20], [21] In contrast, the OR, which is formed by axons of neighboring, more proximal neurons located in the lateral geniculate nucleus (LGN), is known to be a frequent site of MS-related inflammatory demyelination. [22] However, for lesions confined to the optic radiations it would require trans-neuronal transmission to reach RGC axons.

Compelling evidence of retrograde trans-neuronal degeneration in the visual pathway has emerged from animal and human studies recently [23], [24] Mehta and Plant [25] reported topographically accurate reduction of RNFL thickness in patients with long-standing occipital lesions, while Cowey et al demonstrated transneuronal retinal ganglion cell degeneration following cortical lesions in both primate species and humans using MRI. [26] Jindahra et al recently demonstrated trans-synaptic retrograde degeneration in the visual system in acquired lesions of occipital cortex [27] and Bridge et al showed that RNFL thinning presents after post-striate lesions. [28] There is also some evidence that trans-neuronal degeneration may cause axonal loss in MS. [29], [30] In 2012 Inigo et al presented data supporting trans-neuronal degeneration in the visual system of MS patients using Diffusion Tensor Imaging. [31] However, while it is tempting to speculate that trans-neuronal transmission of damage from LGN to RGC may play a part in the observed loss of RGC axons, the cross-sectional nature of the study prevents us from drawing definite conclusions.

It deserves mention that a possible causative association between OR lesions and RGC axonal loss is consistent with the preferential damage of tRNFL fibers supplying the central part of the visual field found in MS patients previously [1], [7], [22], [32], [33] and confirmed by the current study. Horton and Hoyt demonstrated that more than 50% of visual cortex is dedicated to central 10 degrees of the retina. [34] This overrepresentation of the central visual field is largely formed at the retinal level and preserved in the OR. [35] Assuming uniform distribution of lesions within the OR, it is likely that OR fibers sub-serving central vision are damaged more extensively, which, in turn, may cause larger damage of the central (temporal) RNFL.

Another key finding of this study is related to the functional assessment of the outer-retina and its association with RNFL and RGC layer thinning. We demonstrated significant delay of the photopic ERG b-wave in our MS cohort and its correlation with RGC axonal and neuronal deficit. While various ERG abnormalities have previously been reported in MS patients, [36], [37] the finding of a significant association between ERG delay and thinning of RGC layer and RNFL is novel.

Delay of the photopic ERG b-wave found in this study is particularly intriguing considering the recent report by Green et al who identified significant neuronal loss and focal reduction of cell density in the inner nuclear layer of MS patients. [36] Since it is believed that delay of the photopic ERG b-wave indicates impaired response of retinal bipolar cells (which constitute major cellular component of the inner nuclear layer), our finding may represent functional counterpart of the inner nuclear layer structural damage found in that study.

Regarding the correlation of the photopic ERG b-wave delay with thinning of RNFL and RGC layer, several potential explanations can be suggested. Both bipolar and RGC may simultaneously be subjected to MS-related primary retinal process of inflammatory or neurodegenerative nature. Alternatively, since bipolar and ganglion cells are in direct contact with each other, primary injury of one cellular layer can cause damage to the neighboring cellular layer via the mechanism of trans-synaptic degeneration. Our recent study suggests that the spread of retrograde trans-synaptic degeneration from RGC to bipolar cells is unlikely. [13] This is also in line with studies of experimental optic nerve axotomy, which failed to show damage of outer-retina. [38], [39] Therefore, it remains to be seen whether a primary damage to bipolar cells initiates anterograde degeneration of RGC or primary retinal process affects both neuronal layers simultaneously.

To quantify relative association of outer retinal dysfunction and posterior visual pathway damage with RNFL and RGL thinning we have also performed multivariate linear regression analysis, which confirmed significant relationship of RGC axonal and neuronal loss in NON-eyes of MS patients with both measures. However, estimated predictive power of the posterior visual pathway damage was considerably larger compare to retinal dysfunction, implying on potentially more important role of MS-related optic radiation damage in RGC loss.

In addition, even for the best correlating tRNFL thickness the model explained less then 50% of the variability. Therefore, other factors may be involved in RGC neurodegeneration. Thus, for instance, while binocular nature of RGC axonal and neuronal loss advocates its retro-chiasmal origin, the possibility of sub-clinical ON at least in some cases cannot be fully excluded. Alternatively, moderate correlation may be related to the fact that the degree of the initial inflammation in the posterior visual pathway (and, as a consequence, the level of the chronic demyelination measured by the latency of the mfVEP, does not fully define the extent of the acute lesional axonal loss.

In conclusion, the results of our study demonstrated association of RGC axonal and neuronal loss in NON-eyes of MS patients with both outer retinal dysfunction and post-chiasmal visual pathway damage. However, only longitudinal study, which is now underway, may help to reveal causative relationship between investigated measures.
